# 30 Hz, Could It Be Part of a Window Frequency for Cellular Response?

**DOI:** 10.3390/ijms22073642

**Published:** 2021-03-31

**Authors:** Olga García-Minguillán, Ceferino Maestú

**Affiliations:** 1 Escuela Técnica Superior de Ingenieros de Telecomunicación, Universidad Politécnica de Madrid, 28040 Madrid, Spain; olga.garcia-minguillan.lopez@alumnos.upm.es; 2 CTB (CTB-UPM) Centro de Tecnología Biomédica, Universidad Politécnica de Madrid, 28223 Pozuelo de Alarcón, Spain; 3 CIBER-BBN Centro de Investigación Biomédica en Red, 28029 Madrid, Spain

**Keywords:** NIR, ELF-EMF, cell viability, VGCCs, LVGCCs

## Abstract

Many exogenous and endogenous risk factors have been proposed as precursors of brain tumors, including the exposure to non-ionizing electromagnetic fields. Nevertheless, there is still a debate among the scientific community about the hazard of the effects produced by non-ionizing radiation (NIR) because conflicting results have been found (number of articles reviewed >50). For that reason, to provide new evidence on the possible effects produced by exposure to NIR, we performed different studies with several combinations of extremely low frequencies, times, and field intensities in tumoral and non-tumoral cells. The results of our studies showed that cell viability was frequency dependent in glioblastoma cells. In fact, our results revealed that a frequency of 30 Hz—or even other frequencies close to 30 Hz—could constitute a window frequency determinant of the cellular response in tumoral and non-tumoral cells.

## 1. Why Should We Study the Effects Produced by Non-Ionizing EMFs?

Nowadays it is impossible to imagine a society unexposed to electromagnetic fields (EMFs) originating from different sources [1]. In fact, the EMFs which are the physical combination of electric and magnetic fields are both naturally created, such as the geomatic field, and artificially made, such as with the fields created by appliances [1].

The two main physical parameters that define EMFs are: the wavelength (λ), which is the distance between two crests, and the frequency (f) which is the number of waves per unit of times [2]. 

**Figure 1:**
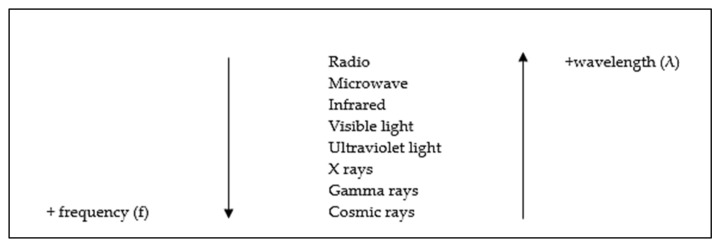
Types of electromagnetic radiation waves that form the electromagnetic spectrum [3].

The set of all EMFs constitute the denominated electromagnetic spectrum, where they are divided depending on their wavelength and frequency into radio waves, microwave, infrared, visible light, ultraviolet light, X-rays, gamma rays and cosmic rays [3] (Figure 1).

The different EMFs are divided into ionizing radiation and non-ionizing radiation by the way that they interact with matter [1,4].

The ability to produce ionization is determined by the energy of the electromagnetic wave and it is related to the frequency of the field [1,4]. In this aspect, ionizing EMFs are those of longer frequencies, e.g., gamma radiation, which have enough energy to produce molecule breakage [1,4]. Meanwhile, non-ionizing EMFs are those of lower frequencies and because of that they do not have enough energy to ionize matter [1,4].

Notwithstanding, many institutions including the World Health Organization (WHO) warm that the exposure to non-ionizing EMFs above some levels could produce biological effects [1,5]. The International Agency for Research on Cancer (IARC), belonging to the WHO, has even classified extremely low frequency (ELF)-EMFs and radiofrequency (RF)-EMFs as group 2B: “possibly carcinogenic to humans” [1,6,7].

Regarding that, several studies have related the exposure to non-ionizing EMFS with the origin or influence of several human pathologies [1,8,9].

Thereupon, many countries have established restriction levels for non-ionizing EMF radiation in order to ensure the highest level of security [1]. 

For example, in Europe, the Council Recommendation 1999/519/EC was published in 1999, in which there were established basic restrictions and recommended levels of exposure. In that recommendation, according to the frequency, the restrictions wereestablished in order to prevent effects on the nervous system (for frequencies between 0 Hz and 10 MHz) or to prevent heating in the tissues (for frequencies bigger than 10 GHz) [1,10].

Generally, exposure limits and recommended levels of exposure are based on the state of the art by scientific organism and international committees [1,11].

Therefore, we consider that when analyzing the effects of non-ionizing EMFs, it is indispensable to narrow down the frequencies of interest and thus selection must consider the applicable legislation [1]. Additionally, it is essential to choose an appropriate study system for the frequencies chosen [1].

Following this approach, we decided to focus our research on the effects produced by ELF-EMFs in a murine model of glioblastoma [1,12,13,14] because the nervous system has been pointed out as one of the most sensitive to EMFs [1,15,16].

Before starting our investigation, we did an exhaustive search about the effects produced by ELF-EMFs in different cell models (Table 1 and Table 2) [1].

Among the different studies performed by other researchers, we would like to highlight the denominated Reflex Project (“risk evaluation of potential environmental hazard from low energy electromagnetic field exposure using sensitive in vitro methods”) because it analyzed the effects produced by EMF of intensities equal or lower to the recommend levels [29].

In fact, the Reflex Project was designed to determine whether EMF exposure could produce hazard effects [29].

For that, in the Reflex Project, they studied both the effect of ELFs and RF-EMFs using different cell types what allowed to check that the effects produced were cell-type dependent [29]. Furthermore, it was observed a possible relation between the effects produced and the exposure parameters [29].

For instance, depending on the type of exposure (continuous or intermittent) different genotoxic and proliferation effects were found [29].

Due to the variability of results, the Reflex Project concluded that the evidence was not enough strong to establish that the current exposure levels were not safe [29].

Nevertheless, based on the hint that the cellular response was dependent on the cell type, prior to starting our experiments, we reviewed the state of the art of specific studies performed with glioblastoma cells models [30,31,32,33,34,35,36,37,38,39].

**Table 2:** Summary of results obtained in different in vitro studies with neuroblastoma and glioblastoma cells lines exposed to ELFs [1,30,31,32,33,34,35,36,37,38,39].

Reference	Experimental Condition	Cell Line	Results
Pessina et al. (2001) [30]	50 Hz, 2000 μT, 24 and 48 h of continuous exposure	U-372	No effects on cell viability
Del Giudice et al. (2007) [31]	50 Hz, 3.1 mT, 18 h of continuous exposure	H4	No effects on cell viability
Koyama et al. (2008) [32]	60 Hz, 5 mT, different times of exposure from 5 h up to 30 h	A172	Any difference in cell survival. No DNA damages were found
Kesari et al. (2016) [33]	50 Hz, 10 µT and 30 µT, 24 h	SH-SY5Y and C6	Any difference in cell viability
Su et al. (2017) [34]	50 Hz, 2 mT, 24 and 48 h of exposure	U251 and A172	No effects on cell viability, cell cycle progression and cell proliferation
Akbarnejad et al. (2017) [35]	50 Hz, 10 mT and 5 mT, different times of exposure (2 h, 4 h and 24 h) 10 Hz, 5 mT, different times of exposure (2 h, 4 h and 24 h) 100 Hz, 10 mT, different times of exposure (2 h, 4 h and 24 h)	U87	↑ number of cells after exposure during 24 h to 10 mT. However, no effect was found after the exposure to 5 mT. ↓ number of cells after 24 h of exposure (10 Hz, 5 mT) ↓ number of cells after 24 h of exposure (100 Hz, 10 mT)
Akbarnejad et al. (2017) [36]	100 Hz, 100 G, different times of exposure (72 h, 96 h, 120 h, 144 h)	U87 and T986	↓ cell viability after 96, 120 and 140 h of exposure. After 72 h of exposure, no significant differences were observed.
Naarala et al. (2017) [37]	18 Hz, 30 μT, 24 h of continuous exposure	C6	No effects on cell viability
Ashta et al. (2020) [38]	50 Hz, 5000 μT, 96 h of continuous exposure	A172	↓ cell viability
Dehghani-Soltani et al. (2020) [39]	50 Hz, 7000 μT different times of exposure (24 h, 48 h, 72 h, 96 h and 126 h)	A172 and and T98	No effects on cell viability

After doing this search, we realized that most studies performed with glioblastoma models varied only time of exposure [30,32,33,34,35,36,39] and almost no one studied the effects produced by different frequencies [30].

In fact, most of them focused only on 50 Hz [30,31,32,33,38,39], probably because it is the utility frequency in Europa and Asia, even though the criteria used to choose the frequency was not described.

Furthermore, we observed that in many of the studies the field intensity used was remarkable greater than the recommended level for ELFs without explaining the reason that led to choosing each intensity [30,31,32,34,35,36,38,39].

Regarding these, although most of them seem to indicate absence of response [30,31,32,33,34], some showed differences in cell viability [35,38].

For instance, after 24 h of continuous exposure to a NIR-EMF of 2 mT at the frequency of 50 Hz, any effects on cell viability were observed in the glioma cell lines U 372 [30], U251 and A172 [34]. In contrast, other research had observed at 50 Hz a decrease in cell viability of A172 after 96 h of exposure to an NIR-EMF of 5 mT [38].

However, the results are not comparable because the exposure parameters were completely different.

**Figure 2:**
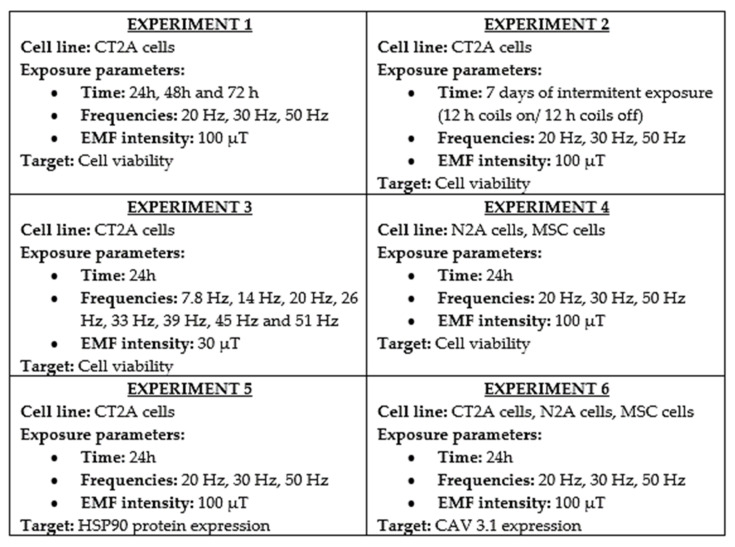
Schematic summary of the experiments designed and performed to archive our goals [1,12,13,14]. In each experiment, the cell line studied is summarized, and the exposure parameters of the experiment and the parameter (target) is analyzed after the exposure.

This situation motivated us to design a study to achieve the following goals (Figure 2) [1]:

(1) Analyze the effect produced by an EMF of intensity equal to the recommend level for ELFs [1,12].
(2) Compare the effects produced by continuous and intermittent exposure to ELF-EMFs [1,13,14].
(3) Study the effect produced by ELF-EMFs of the intensity equal to those generated by the Earth [1,13,14].
(4) Determine if ELF-EMFs could produce micro-thermal effects [1,13,14].
(5) Compare the effects produced by ELF-EMFs on cell viability of a glioblastoma model to the effects produced in other tumoral and non-tumoral cell lines [1].
(6) Study calcium-voltage gated channel type T as a possible biomarker of cell response [1].

## 2. The Frequency of Exposure, an EMF Parameter That Cannot Be Ignored in Cellular Response

Current legislation in the European Union has not established restrictions for the time exposure and other critical field parameters such as the frequency or the wave form [1,10].

Motivated by this legal vacuum, we decided to investigate the effects produced by a fixed intensity extremely low frequency (ELF)-EMF on a glioblastoma cell line after exposure to different frequencies and times [1].

For that, we first designed a multi-assay where we continuously exposed CT2A mouse glioma cells to a 100 µT EMF during 24, 48 and 72 hours to three different ELFs: 20, 30 and 50 Hz [1,12].

The ELF exposure system was set up by a pair of two identical coils (19 turns, 12 cm of radius) separated by a distance equal to their radius in a Helmholtz configuration [1,12].

The intensity of the field was chosen according to the exposure level suggested by the Council Recommendation 1999/519/EC, for frequencies between 8 and 25 Hz (i.e., 5/frequency Teslas) [10] and controlled by a function generator (FAC 662B) and a power supply (Frederiksen) [1,12].

**Figure 3:**
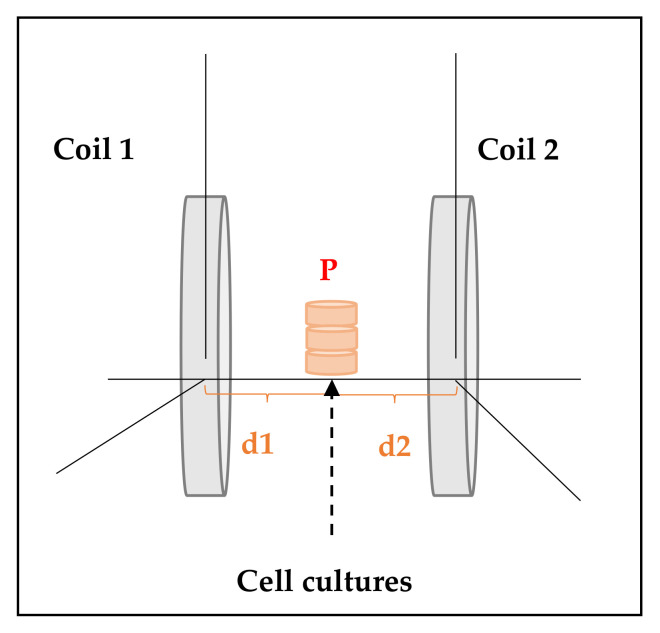
ELF-electromagnetic field (EMF) exposure system’s scheme. For each experiment, three culture dishes (diameter = 35 mm) were placed in a point P equidistant to coil 1 and coil 2 (i.e., distance d1 from point P to coil 1 was equal to distance d2 from point P to coil 2) [1].

For each experiment, three culture dishes were placed in the middle of the axis of the coils (Figure 3) and the homogeneity of the field was checked prior to the begging of the experiments by a gaussemeter (LakeShore-Cryotronics 450) [1,12].

In order to guarantee the adequate physiological condition for the cultures (i.e., 37 °C, 5% CO_2_ and 95% humidity) the pair of coils were kept inside a cell incubator and isolated from external EMF by a mu-metal box [1,12].

The control group (unexposed) was kept in the same condition without exposure (coils off) and all the experiments were replicated three times [1,12].

In our opinion, for in vitro studies, the isolation of the cultures from external EMFs is a key step because the fan of the incubator produces an EMF at a given frequency [1]. It means that, if you design an experimental set without a proper shielding, the control groups would not be truly unexposed (0 Hz) and therefore, the comparison with the exposed groups would be corrupted [1].

Another critical aspect in the design of these experiments is the selection of the study parameter and the method to evaluate it [1]. 

In our case, we first focused on the effects produced on cell viability [1,12] because it is a measurement of the proportion of living cells, and it allows the optimization of the experimental conditions [40]. 

There are different methods available to analyze cell viability which can be divided mainly into permeability assays and metabolic assays [1,40].

We decided to study the effects of ELF-EMFs on cell viability through colorimetric assay XTT [1,12], which is a metabolic assay based on the reduction of trazolium salts [1,41].

For that, immediately after the exposure each study group and the control groups were incubated with XTT for 1 h in the dark at 37 °C and 5% CO_2_ [1]. Next, we measured the absorbance of the samples with a spectrophotometer and we computed the % of cell viability as follows (Equation (1)) [1]:

(1) $$\%\;of\;cell\;viability=\frac{Group\;Experiment\;absorbance}{Control\;group\;absorbance}\times \;100\;\%$$

Equation (1) % of cell viability. For the control group, the absorbance measured was considered equal to 100% of cell viability [1].

**Figure 4:**
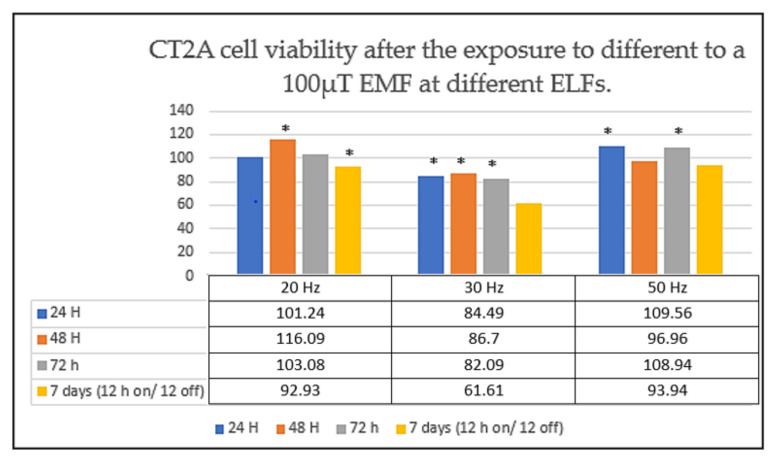
CT2A cell viability after the exposure to different non-ionizing EMFs. CT2A cells were exposed to a 100 µT EMF during 24 h (continuous exposure), 48 h (continuous exposure), 72 h (continuous exposure) and 7 days (intermittent exposure: 12 h coils on/12 h coils off) to three ELFs: 20, 30 and 50 Hz. The data are expressed as the means ± standard error, and * indicates a significant cell viability difference from the CT2A control group (*p* < 0.05) [1,13,14].

Through this experiment we found that the cell viability of CT2A glioma cells was dependent on the frequency but not on the time of exposure (Figure 4) [1,12]. 

In fact, we observed that the number of CT2A cells appeared to increase at 50 Hz, while at the frequency of 30 Hz we observed a significant cell viability reduction (Figure 4) [1,12].

No morphological changes were associated to the difference in cell viability observed between the control group and the cell exposed [1]. 

From these findings, we decided to design a second experiment to analyze whether an intermittent ELF-EMF could produce a similar effect on CT2A cells [1,13,14].

Specifically, in the second experiment we exposed CT2A for 7 days to cycles of 12 h of exposure followed by 12 h without exposure [1,13,14]. For that, a timer plug was added to the experimental set up [1,13,14].

Strikingly, we observed that an intermittent EMF of 100 µT at 30 Hz also produced a notable decrease in the number of CT2A cells (Figure 4) [1,13,14]. Whilst at the frequency of 50 Hz we did not obtain any difference between the control groups (cells unexposed) and the CT2A cells exposed (Figure 4) [1,13,14].

Nevertheless, we have the hypothesis that CT2A cells did respond to the intermittent exposure at 50 Hz but due to the long duration of the experiments it could produce a nutrient depletion [1].

On one hand, to avoid nutrient depletion and accumulation of toxic wastes, it is essential to renovate the culture with fresh medium [1,42]. On the other hand, to guarantee a proper cell growth, it is necessary to divide the cellular content in other culture plates or flask [1,38].

Therefore, to avoid possible false negatives due to lack of nutrient exhaustion we limited our next experiments to 24 hours of exposure [1,13,14].

Following this approach, the next point we addressed was the study of the effects produced by an EMF of only 30 µT [1,13,14], equal to the lowest intensity of the field generated by the Earth [43] at the different frequencies of the Schumann resonance [1,13,14,44].

For that, we compared the cell viability of the CT2A control groups with the total number of viable cells after the exposure to 7.8, 14, 20, 26, 33, 39, 45 and 51 Hz [1,13,14,44].

In this experiment, it was remarkable that CT2A cells seemed to be sensitivity to a 30 µT at almost all the frequencies [1,13,14]. Particularly, CT2A cells seemed to be extremely sensitive to 33 Hz where we observed a cell viability decrease of around 40% in comparison with the control group (cells unexposed) [1,13,14].

This finding made us ask two questions:

(1) First, could be CT2A cells especially sensitive to a frequency range close to 30 Hz [1]? and,
(2) Second, could this range of frequencies cause a similar response in other cell lines [1]?

Thus, we proposed a complementary study in which we exposed for 24 h two different types of cells: mouse mesenchymal stem cells (MSC) and N2A murine neuroblastoma cells [1].

For that, MSC and N2A cells were exposed to a continuous EMF of 100 µT at 20, 30 and 50 Hz by using the exposure system described before [1].

Then, we focused on analyzing the effects produced by these non-ionizing EMFs in terms of cell viability, and we compared the results with the response observed previously in our first sets of experiments with CT2A cells [1].

**Figure 5:**
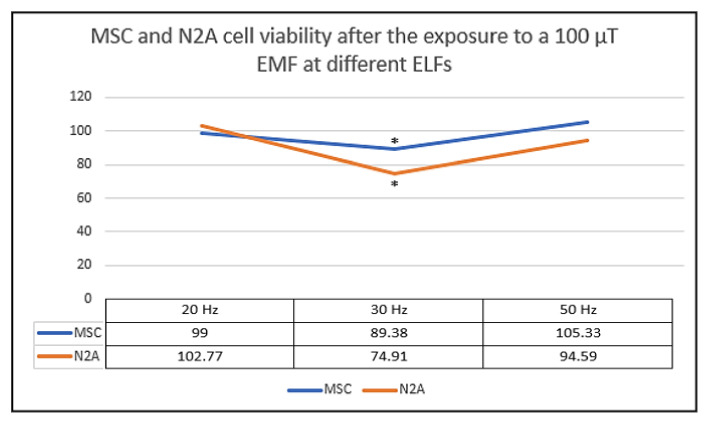
Mesenchymal stem cells (MSC) and N2A cell viability after 24 h exposure to a non-ionizing field of 100 µT. The results from three independent experiments which each frequency and cell type are plotted on graph. The data are expressed as the means ± standard error, and * indicates a significant cell viability difference from MSC and N2A cells to their corresponding control groups (*p* < 0.05) [1].

Here, it is important to highlight that both MSC and N2A cells were very sensitive to an EMF of 100 µT at the frequency of 30 Hz (Figure 5) [1]. In fact, both cell types showed a significant cell reduction at 30 Hz which is a cellular response similar to the cell viability decrease observed in CT2A cells at that frequency [1]. 

## 3. HSP90 Protein Is Not Suitable Biomarker in Non-ionizing Studies with ELF-EMFs

Heat shock proteins (HSP) constitute a family of proteins that are activated by cell damages [45]. That means, in normal conditions, HSPs remain inactivated and under a stressful cell situation they become activated to act as a protection mechanism [1,45].

In the past, some in vitro studies described an increase of HSPs expression in different cell types after the exposure to ELF-EMFs [45].

Although non-ionizing ELF-EMFs do not have enough energy to produce heat damages [4], we considered micro-thermal effects as a possible explanation for the differences observed in the cell viability [1,13,14].

Among the different types of HSPs, HSP90 has been related to different pathologies [45] and its inhibition in tumor cells has been reported [46].

Then, we decided to analyze if a 100 µT ELF-EMFs could produce an increase of HSP90 protein expression in CT2A cells [1,13,14].

For that, after the exposure during 24 hours to 20, 30 and 50 Hz we compared by indirect immunofluorescence the expression of HSP90 protein in the exposed cells with the expression of that protein in the control groups (CT2A unexposed) [1,13,14].

To rule out false negative we perform a preliminary study without exposure where we varied the temperature of the culture from 37 °C up to 39 °C. Here, we observed HSP90 expression increased in the CT2A cells maintained at the biggest temperatures (i.e., 38 °C, 38.5 °C, and 39 °C) [1,13,14].

However, when analyzing the effect of exposure to ELF-EMFs no difference was observed. Namely, our results revealed that a 100 µT EMFs did not produce any change in the expression of that protein in the CT2A exposed cells in comparison with the expression on HSP90 protein in unexposed CT2A and maintained under normal physiological conditions (37 °C and 5% CO_2_) [1,13,14].

## 4. Type T Voltage-gated Channels, a Potential Mediator of Cell Response to EMF Stimuli

Calcium ion is a critical cellular response mediator as it is involved in many essential cellular processes such as cell proliferation, gene transcription or apoptosis [1,47,48,49]. Therefore, calcium ion concentration is highly regulated through different mechanism, including transmembrane proteins [1,49].

In tumoral cells, an aberrant calcium movement has been shown which in many situations involved abnormal expression of calcium transporter proteins [49]. One of those calcium transporter proteins are the voltage gated calcium channels (VGCCs) which allows the flux of calcium ion across the cell membrane [50].

Up to now, 5 different types of VGCCs have been described: L, P/Q, N, R, T [47] (Table 3).

All VGCCs need strong depolarization to open, except T-type channels which are low voltage activated and for that reason they are often referred to as “low voltage-gated calcium channels” (LVCCs) [51].

In the past years, an abnormal expression has been observed in the different isoforms of LVCCs (cav3.1, cav3.2 and cav3.3) in several types of tumor [47,48] including those from the nervous system [49].

This fact, together with some in vitro studies which have found that treatment with Mibefradil, a specific T-type channel inhibitor, resulted in a significant cell viability decrease and a furtherance of apoptosis, has pointed out LVCCs as promising therapeutic targets [52].

However, are LVCCs only pharmacological therapeutic targets or could they also be selectively inhibited by other stimuli? [1]

This question has hardly been asked, but we believe it should not be disregarded. In the 80s, a theory was proposed to explain the cellular response induced by NIR-EMFs, in which NIR would be translated by our organism and turned into an afferent signal [53,54].

Other researchers have established that NIR-EMFs are able to interact with the cell membrane leading to a calcium release [55]. 

Nowadays, various hypotheses are focused on VGCCs and in the specific case of ELF-EMFs they are focused on LVCCS [56,57]. In fact, we have the hypothesis that ELF- EMFs could somehow inhibit LVGCCs as they are susceptible to little current changes [1].

For that reason, we analyzed in CT2A, N2A and MSC cells the expression of one of the isoforms of type T VGCCs, cav 3.1 [1]. 

It is important to clarify that, the aim of our experiment was not to study the opening of VGCCs isoform but its pattern expression [1]. We study it by indirect immunofluorescence with anti-CACNA1G antibody [1].

Here, according to our results cav 3.1 protein is positively expressed in the cell membrane of both N2A and CT2A cells, meanwhile in MSC cells, cav 3.1 seemed to be expressed only in the membrane of damage cells [1].

Up to now, the mechanism by which VGCCs are expressed in stem cells has not been well described. For instance, some researchers did not detect cav 3.2 protein in bone marrow stem cells until the third day of cellular differentiation [58].

Therefore, type T LVCCs could be a potential mediator of cell response to EMF stimuli in only some cell types, such as glioblastomas and neuroblastomas cells [1].

Another major complication is that there is still not an agreement about cellular response induced by NIR-EMF and how it is moderated.

## 5. Conclusions

Our results show that, although there are evidences that each cell type has a specific response capacity to EMF, it could be the range of frequencies close to 30 Hz that produce a decrease in the cell viability of different cell lines [1,12,13,14]. Without any doubts, that is the most outstanding finding of our years of research—due to its possible clinical applications. In the literature, this frequency has barely been researched or intended for specific applications.

However, based on our results, we believe there is a moderating frequency close to 30 Hz which triggers the effects produced on cells after the exposure [1]. The question is, how could this modulation be produced?

In the 80s it was described as a potential “frequency windows effect” according to which some frequencies could produce changes in calcium influx [59,60].

Additionally, in the 80s, a mathematical model of ion cyclotronic resonance was also proposed through which it was described that an EMF at a proper combination of intensity and frequency could produce changes in the ion flux [61].

Based on current knowledge and guided by our results, we think that non-ionizing EMF can interact with cell membranes at a certain frequency producing a change in the opening of VGGC type T what would lead to changes in calcium concentration [1]. 

Nonetheless, more in vitro studies are required to confirm this finding, because currently there are hardly any results with frequencies close to 30 Hz [1]. Thus, this is the main limitation of our study and for that, we encourage the development of a bulk of complementary studies using frequencies between 30 to 39 Hz.

Another limitation of our research is that we studied the effects on three types of cell lines exposed mainly to 100 µT. Accordingly, to corroborate our results, future studies should be extended to other tumoral and non-tumoral cell lines using different durations and EMFs of several intensities [1].

## Figures and Tables

**Table 1:** Summary of results obtained in the past 15 years with tumoral, and non-tumoral cells lines exposed to different extremely low frequencies (ELFs) [1,17,18,19,20,21,22,23,24,25,26,27,28].

Reference	Experimental Condition	Cell Lines	Results
Wolf et al. (2005) [17]	50 Hz, 500 µT, 750 µT and 1000 µT, 24h, 48 h and 72 h of continous exposure	HL-60 WI-38 Rat-1	↑ cell proliferation and cell damage
Vianale et al. (2008) [18]	50Hz, 1000 µT, from 24 h to 96 h of continous exposure	Human queratinocites	↑ cell growth
Chen et al. (2010) [19]	60 Hz, 1200 µT, 72 h of continous exposure	HeLa	↓ cell proliferation
Trillo et al. (2012) [20]	50 Hz, 100 µT, 42 h of intermitent exposure	NB69 cells HepG2	↑ cell number
Martínez et al. (2012) [21]	50 Hz, 100 µT, 63 h of intermitent exposure	NB69 cells	↑ cell number
	50 Hz, 100 µT, 63 h of continous exposure		No effects on cell viability
Cid et al. (2012) [22]	50Hz, 10 µT, 24, 42 y 92 h of intermittent exposure	HepG2	↑ cell number
Mo et al. (2013) [23]	Hipomagnetic field (≤50 µT)	SY5Y	↑ cell viability
Destefanis et al. (2015) [24]	50 Hz, 45 µT, 7 days of continuous exposure	SKBR3 GTL16	Cell growth inhibition
Yuanet al. (2017) [25]	50 Hz, 5100 µT, 30 min, 1 h and 2 h of exposition during 3 days	G401 N2A CHLA255	↓ cell viability
Nasrabadi et al. (2018) [26]	50 Hz, 1000 µT, 8 h of exposure during 3 days	hRPE	No effects on cell proliferation
Tang et al. (2019) [27]	7.83 Hz, 300 µT, 24h and 48 h of continous exposure	B16F10	↓ cell viability
Chenet al. (2020) [28]	50 Hz, 400 µT, 15 min, 30 min, 1 h and 24 h	FL cells	Cell proliferation promotion

**Table 3:** Summary of voltage gated calcium channel (VGCC) isoforms: list of genes that codifies the different proteins that constitute the VGCCs [48].

VGCCs	Protein (Gen)
L-type calcium channel	Cav 1.1 (*CACNA1S*) Cav 1.2 (*CACNA1C*) Cav 1.3 (*CACNA 1D*) Cav 1.4 (*CACNA1F*)
P/Q-type calcium channel	Cav 2.1 (*CACNA1A*)
N-type calcium channel	Cav 2.1 (*CACNA1B*)
R-type calcium channel	Cav 2.3 (*CACNA1E*)
T-type calcium channel	Cav 3.1 (*CACNA1G*) Cav 3.2 (*CACNA1H*) Cav 3.3 (*CACNA1I*)
